# Evaluating data integrity in ribosome footprinting datasets through modelled polysome profiles

**DOI:** 10.1093/nar/gkac705

**Published:** 2022-08-18

**Authors:** Fabio Hedayioglu, Emma J Mead, Patrick B F O'Connor, Matas Skiotys, Owen J Sansom, Giovanna R Mallucci, Anne E Willis, Pavel V Baranov, C Mark Smales, Tobias von der Haar

**Affiliations:** Kent Fungal Group, School of Biosciences, Division of Natural Sciences, University of Kent, Canterbury CT2 7NJ, UK; Industrial Biotechnology Centre, School of Biosciences, Division of Natural Sciences, University of Kent, Canterbury CT2 7NJ, UK; School of Biochemistry and Cell Biology, University College Cork, Cork, Ireland; Kent Fungal Group, School of Biosciences, Division of Natural Sciences, University of Kent, Canterbury CT2 7NJ, UK; Cancer Research UK Beatson Institute, Garscube Estate, Switchback Road, Glasgow G61 1BD, UK; Institute of Cancer Sciences, University of Glasgow, Garscube Estate, Switchback Road, Glasgow G61 1QH, UK; UK Dementia Research Institute at the University of Cambridge and Department of Clinical Neurosciences, Island Research Building, Cambridge Biomedical Campus, Cambridge CB2 0XY, UK; MRC Toxciology Unit, University of Cambridge, Tennis Court Rd, Cambridge CB2 1QR, UK; School of Biochemistry and Cell Biology, University College Cork, Cork, Ireland; Industrial Biotechnology Centre, School of Biosciences, Division of Natural Sciences, University of Kent, Canterbury CT2 7NJ, UK; Kent Fungal Group, School of Biosciences, Division of Natural Sciences, University of Kent, Canterbury CT2 7NJ, UK

## Abstract

The assessment of transcriptome-wide ribosome binding to mRNAs is useful for studying the dynamic regulation of protein synthesis. Two methods frequently applied in eukaryotic cells that operate at different levels of resolution are polysome profiling, which reveals the distribution of ribosome loads across the transcriptome, and ribosome footprinting (also termed ribosome profiling or Ribo-Seq), which when combined with appropriate data on mRNA expression can reveal ribosome densities on individual transcripts. In this study we develop methods for relating the information content of these two methods to one another, by reconstructing theoretical polysome profiles from ribosome footprinting data. Our results validate both approaches as experimental tools. Although we show that both methods can yield highly consistent data, some published ribosome footprinting datasets give rise to reconstructed polysome profiles with non-physiological features. We trace these aberrant features to inconsistencies in RNA and Ribo-Seq data when compared to datasets yielding physiological polysome profiles, thereby demonstrating that modelled polysomes are useful for assessing global dataset properties such as its quality in a simple, visual approach. Aside from using polysome profile reconstructions on published datasets, we propose that this also provides a useful tool for validating new ribosome footprinting datasets in early stages of analyses.

## Graphical Abstract

**Graphical Abstract:**
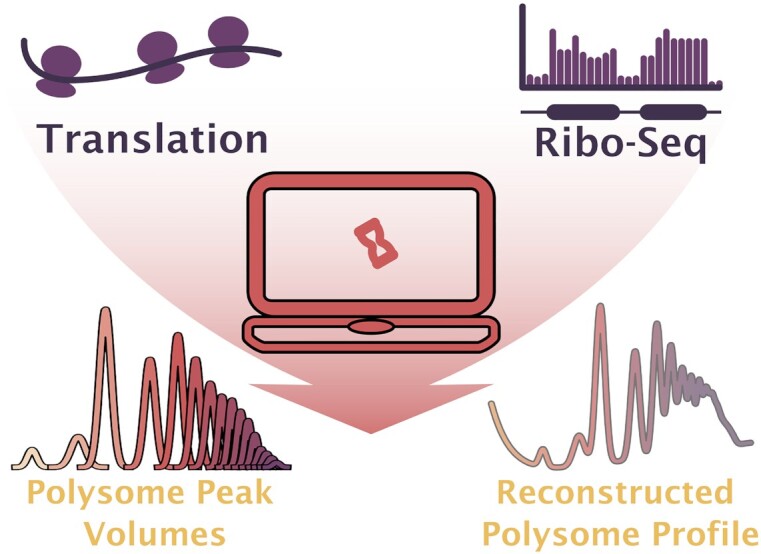
Projection of Ribo-Seq datasets into the graphical format of simulated polysome profiles allows rapid investigation of the sequencing data at intermediate resolution.

## INTRODUCTION

During protein synthesis, mRNAs are typically decoded by multiple ribosomes simultaneously (1). The average ribosome load per mRNA is a useful proxy for assessing transcriptome-wide translational activity (2) and the relative balance between translation initiation and elongation rates (3–5). This parameter can be accessed by two principal experimental techniques which operate at different levels of resolution, namely polysome profiling and ribosome footprinting (also termed ribosome profiling or Ribo-Seq).

**Figure 1.**
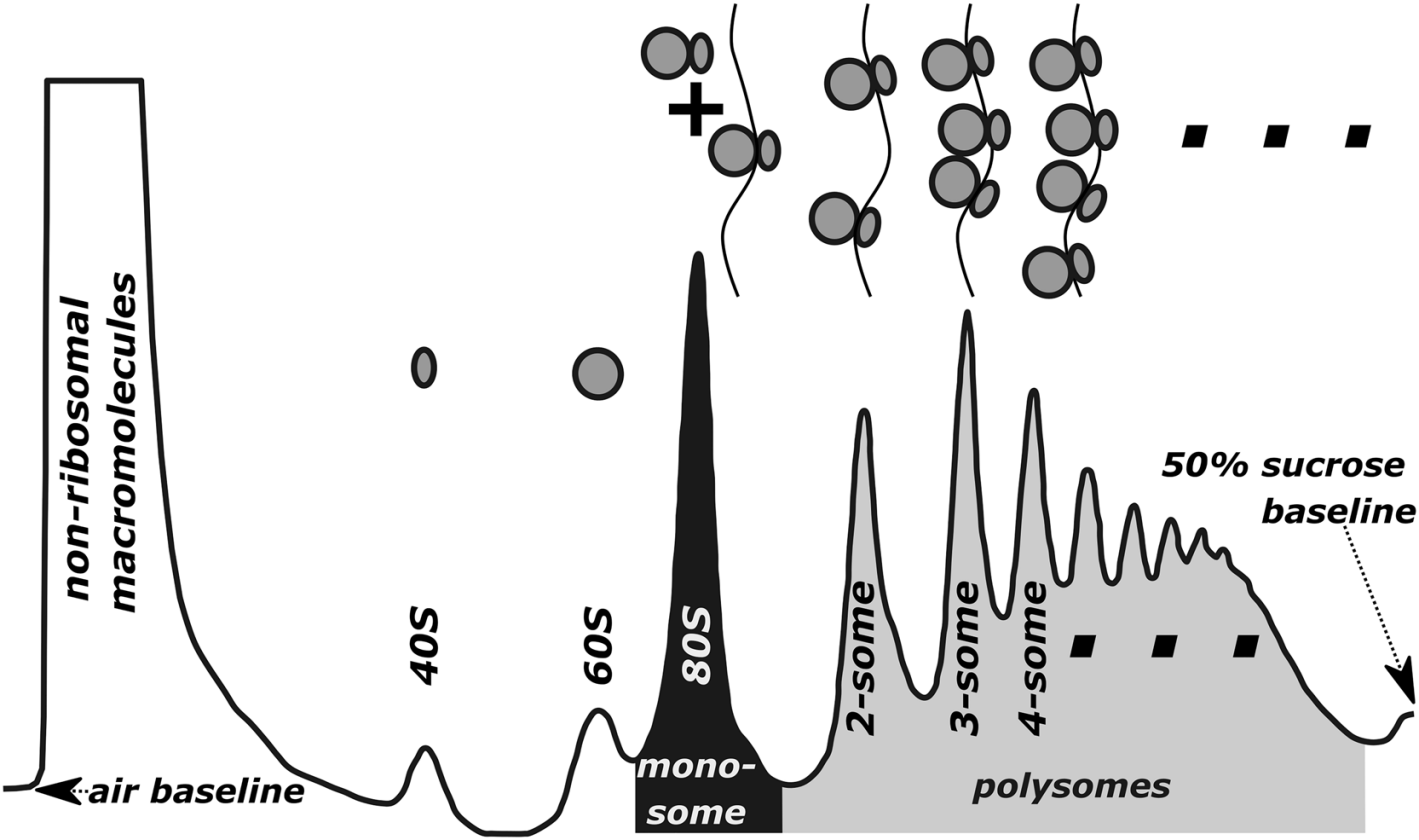
Components of a polysome profile. The representative profile was generated with cycloheximide-arrested polysomes from logarithmically growing *S. cerevisiae*, centrifuged through a 7–47% sucrose density gradient followed by optical density scanning at 254 nm wavelength. Selected peak locations are indicated. The shaded areas correspond to the ‘monosome’ (black) and ‘polysome’ (light grey) regions of the profile, which are frequently used to calculate the P/M ratio as a proxy for translational activity.

Polysome profiling is a long-established method for assessing transcriptome-wide ribosome loads. Widely used in particular for assessing eukaryotic translation, it relies on sucrose density gradients (6), on which populations of mRNAs bound to different numbers of cycloheximide-arrested ribosomes can be separated into individual peaks (7,8) (Figure 1). Polysome profiles are often evaluated qualitatively by assessing the relative height of monosome versus polysome peaks, or semi-quantitatively by calculating a polysome/monosome (P/M) ratio based on the areas under the corresponding peaks (indicated as shaded areas in Figure 1).

The information content of polysome profiles is contained in several parameters, including the relative peak location which corresponds to the number of ribosomes bound to an mRNA, and relative peak height which for the *n*th peak corresponds to the combined mRNA, rRNA and tRNA content of all transcripts translated by *n* ribosomes (an ‘n-some’), where *n* is counted from the monosome peak onwards. In *Saccharomyces cerevisiae* each ribosome contains around 6000 nucleotides of rRNA (9) whereas the average transcript length is 1600 nt (10), and rRNA absorbance therefore greatly dominates mRNA absorbance in all polysome peaks. Similar, albeit less extreme rRNA:mRNA ratios, are also observed in higher eukaryotes.

Ribosome footprinting is a more recently developed approach where the location of individual ribosomes on transcripts is determined via deep sequencing of RNA fragments generated when bound ribosomes protect transcripts from nuclease digestion (11,12). With this technique the information content is contained in the returned sequences, which align to the location of the bound ribosome in the transcript sequence, and in the frequency with which each sequence occurs in the mixture. The latter can be evaluated at different levels. At one level, the frequency of sequences originating from one transcript versus those originating from another is proportional to the relative ribosome loads of the two transcripts. At another level, the distribution of sequences within the same transcript is inversely proportional to the speed of ribosomes traversing each section of the transcript.

Polysome profiling and ribosome footprinting experiments can be considered to contain overlapping sets of information. Ribosome footprinting reveals more detailed information as it allows assessing translational activity of individual sequences, whereas polysome profiles only reveal summative information on the relative abundance of groups of sequences with similar ribosome loads. Thus, a polysome profile can be viewed as a subset of the information within a ribosome footprinting experiment, with the exception that information on non-translating ribosomes, which is present in the 40S, 60S and 80S peaks of a polysome profile, is not represented in ribosome footprinting experiments.

Ribosome footprinting datasets tend to be interpreted in a very detailed manner, usually including comparisons of ribosome densities between individual genes, and in some cases also of ribosome densities on individual codons. Such detailed analyses are sometimes complemented by metagene analyses, where footprint densities are averaged over all genes or subsets of genes contained in an organism. Compared to these two extremes, polysome profiles have an intermediate information content—they reveal a more detailed breakdown of translational activity than metagene analyses, but summarise information more than gene-level footprint analyses. We reasoned that interpreting footprinting datasets at the information level of polysome profiles could add significant insights to current analyses of footprinting data. Moreover, details of the footprinting methodology are still being developed, in particular with respect to the use of cycloheximide during the initial preparation of cell extracts (13–15), but also with respect to library preparation (16), the nucleases used to generate the ribosome protected fragments (17), and other parameters (18–20). A simple visual way of examining the integrity of ribosome footprinting datasets would provide a useful tool to assess newly generated datasets.

Herein, we describe the development of methodology and software for simulating polysome profiles from ribosome footprinting datasets. We apply this methodology to published ribosome footprinting datasets from yeast and mammalian cells and reveal that there is indeed a correspondence between polysome profiles and Ribo-Seq data, but we also identify heterogeneity in the apparent proportion of very heavily ribosome bound mRNAs. In many datasets, an apparent excess of heavy polysomes is corrected when higher quality reference data for mRNA expression are used, whereas in a smaller subset of datasets the excess of heavy polysomes persists even when evaluated with reference mRNA data. Further investigation of this subset of datasets yielding non-physiological modelled polysome profiles demonstrates that such datasets display other issues, such as generally having a poor correlation of transcript-specific ribosome densities with other datasets, and low base-quality scores for the Ribo-Seq data. Overall we demonstrate that modelled polysome profiles are a convenient visual tool for assessing RiboSeq dataset integrity, and we provide the polysome modelling functionality as a simple to install python package.

## MATERIALS AND METHODS

### Polysome profiles

Polysome profiles were generated using sucrose density gradients as described (21). The *Saccharomyces cerevisiae* profile was generated using yeast strain BY4741 (22) transformed to URA^+^ and grown to OD_600_ of 0.62 at 30°C in defined (SC) medium lacking uracil. The mammalian cell profile was generated using HEK293 cells grown to 70% confluence under standard conditions in Dulbecco's modified Eagle's medium and supplemented with 10% heat-inactivated fetal bovine serum. Cell lysates for sucrose density gradients were generated as previously described (23).

### Computational tools

All analyses were performed using Python 3.7.6 (Python Software Foundation) in Jupyter Notebooks (24). Libraries used included Numpy (25), SciPy (26), SciKit Learn (27), Matplotlib (28) and Pandas (29). All data and Python scripts are available from GitHub (https://github.com/tobiasvonderhaar/polysomes). The software for generating and comparing modelled polysomes has been published using the Python Packaging Index (PyPI, https://pypi.org/project/polyan/) and is freely available at no charge.

### Determination of peak locations in polysome profiles

Initial sets of peak locations were determined either by visual inspection of polysome profiles, or computationally by identifying profile regions where four or more consecutive points with monotonically increasing OD_254_ were followed by four or more consecutive points with monotonically decreasing OD_254_. Peak locations identified either way were then fitted to a logarithmic function, using species-specific fractional polysome numbers for the 40S and 60S peaks, respectively (the fractional number for the small subunit being 0.35 for yeast and 0.37 for mammals, based on the known relative masses of the small and large ribosomal subunits). Additional peak locations were then calculated based on a fitted logarithmic trendline as described.

### Polysome modelling

Individual peaks in the polysome profile were modelled based on scaled probability density functions (PDFs) of a normal distribution:

$$\begin{equation*}{\rm{f}}\left( {{\rm{x}}|{\rm{\mu }},{\rm{\sigma }},{\rm{s}}} \right) = \frac{1}{{\sqrt {2{\rm{\pi }}{{\rm{\sigma }}}^2} }} \cdot {{\rm{e}}}^{ - \frac{{{{\left( {{\rm{x}} - {\rm{\mu }}} \right)}}^2}}{{2{{\rm{\sigma }}}^2}}} \cdot {\rm{s}}\end{equation*}$$

where μ is the location of the peak's maximum, σ its standard deviation, and s is a scaling factor to adjust the area under the peak to the experimental data. The OD contributions from the individual peaks were then summed to generate the complete profile. Contributions from initial debris peaks and from drifting baselines were modelled as an exponential decay function and a linear function, respectively, and were added to the summed PDFs.

### Ribosome footprinting datasets

Ribosome footprinting datasets for yeast were retrieved from the NCBI GEO database (30) (https://www.ncbi.nlm.nih.gov/geo/) using the search term ((‘Saccharomyces cerevisiae’[ORGN]) AND (‘high throughput sequencing’[Platform Technology Type]) AND ribosome). Returned results were manually screened for such datasets that reported footprinting data, or corresponding footprinting and RNA-Seq data, for standard laboratory yeast strains grown in YPD or SC at 30°C, either formatted as counts per gene, counts per codon, or RPKM. All datasets were reformatted to counts per gene before further computational processing, based on gene length information for the R64-2-1 release of the yeast genome. Datasets for HEK293 cells were identified using a similar strategy but replacing the organism specification in the search term with ‘HEK293’.

### Modelling polysome profiles from ribosome footprinting datasets

Relative ribosome densities were calculated for each individual gene as follows: RNA abundances were calculated by converting RNA counts to RPK, and assuming a total cellular RNA number of 60 000 for yeast (31) or 300 000 for HEK293 cells. The number of ribosomes bound to RNAs corresponding to the same gene were calculated from its footprint counts, assuming a total ribosome number per yeast cell of 200 000 of which 85% are active during fast growth in rich medium (31). In HEK293 cells, we assumed a total ribosome content of 2 million. The average ribosome density was then calculated from these values, and the ribosomes assigned to polysome peaks corresponding to the integers on either side of the average density. For an example transcript expressed at 10 RNAs per cell each carrying 5.8 ribosomes on average or 58 ribosomes in total, 11.6 ribosomes (=20% of 58) would be assigned to the five-some peak and 46.4 ribosomes (=80% of 58) to the six-some peak.

Following assignment of RNA-bound ribosomes to their n-some peaks, the remaining, inactive ribosomes (15%) were assigned to the monosome, 60S and 40S peaks. For control conditions and fast growing cells, the proportion of inactive ribosomes was assumed to be 15%, and it was also assumed that 3 out of 10 of these inactive ribosomes were split into separate subunits (this generated the best fit to experimental profiles). For figures where polysome profiles were modelled without inclusion of inactive ribosomes, this step was omitted by setting the *include_idle* parameter passed to the modelling function to *False*.

### Cluster analyses

To assess the similarity between modelled yeast polysomes, peak volumes for each dataset were tabulated and subjected to a hierarchical clustering approach (32). The Euclidean distance between clusters was calculated using the scipy linkage function with the ‘complete’ method, which assigns distances based on the farthest point between clusters. The optimal number of clusters was determined by maximising the Davies–Bouldin index (33), the ratio of between-cluster similarity distances and within-cluster similarity distances.

### RMSD-based *P*-values

Based on the analyses shown in Figures 5–7 a reference ‘*known* good’ collection of yeast datasets was established which showed low RMSD values compared to the experimental profile and where our analyses did not reveal any other issues with data quality. This reference collection comprised datasets GSE106572, GSE107718, GSE109343, GSE109734, GSE116523, GSE122039, GSE124428, GSE13750, GSE34082, GSE41590, GSE45366, GSE50049, GSE51164, GSE51532, GSE52119, GSE53313, GSE56622, GSE59573, GSE61753, GSE63789, GSE66411, GSE67387, GSE72030, GSE76117, GSE81269, GSE84746, GSE85036, GSE85198, GSE85590, GSE86466 and GSE87614. RMSD values for all possible pairwise combinations of datasets were calculated using the yeast reference RNA profile. The parameters of the best fitting normal distribution to the resulting RMSD values were estimated using the stats.norm functionality in scipy. *P-*values are calculated as the probability of observing a given RMSD value in a normal distribution with the fitted parameters.

### Determination of Kulback–Leibler divergence

Raw datasets were downloaded from the sequence read archive (34) and processed as described by adapter trimming, alignment to the yeast genome and analysis using the RUST suite of Python tools (35). The KL-value at codon 40 of the processed aligned reads was used as A-site specific value, whereas the maximum KL value in the region 30–37 (i.e. 3–10 codons upstream from the A-site) as used as the 5′-KL value, and the maximum in the region 43:50 (3–10 codons downstream of the A-site) was used as the 3′-KL value.

### Statistical analyses

To test for statistical significance of differences in dataset parameters in Figure 7A, two-tailed t-tests were conducted comparing parameter values for the main dataset cluster with parameters for the separately clustering datasets (all for analyses conducted with the external reference mRNA data). The *P*-values reported in Figure 7 were adjusted for multiple testing using Holm–Sidak adjustment. Decision tree analyses were conducted using the SciKitLearn implementation of the Extremely Randomised Trees classifier (36) with 100 estimators (the reported results are robust to changing the numbers of estimators in the range from 10 to 1000).

## RESULTS

### Quantitative features of polysome profiles

**Figure 2.**
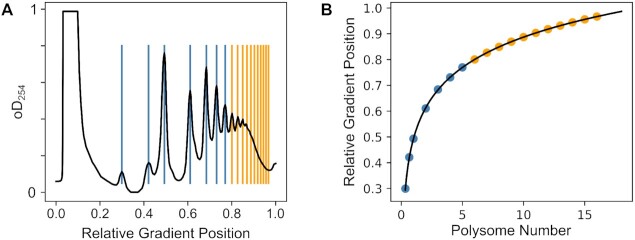
Peak locations in polysomal gradients. (**A**) Peak locations in polysome profiles generated with logarithmically growing yeast cells. Blue lines indicate peak locations detected directly using a peak finding algorithm, orange lines indicate peak locations extrapolated from the directly detected peak locations. (**B**) Peak locations (coloured dots, colours corresponding to panel A) compared to the prediction function (black line).

Interpreting experimental polysome profiles can be problematic since peaks corresponding to monosomes and lower number polysomes are often easily visually distinguishable, but higher polysome peaks are obscured due to their smaller size and closer spacing (Figure 1). However, the location of mass densities in zonal gradients follows known mathematical rules (37), so that peak locations in the denser gradient region can in principle be predicted based on locations of the well separated peaks. In practice, we found that in well-formed gradients peak locations can be accurately related to polysome numbers using an exponential function (Figure 2). This holds true for both polysome gradients generated with yeast (Figure 2) and HEK293 cells (Supplemental Figure S1).

**Figure 3.**
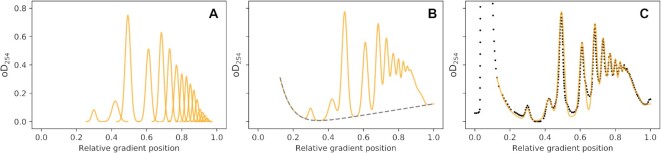
Approximation of a yeast polysomal gradient profile as a Gaussian mixture model. (**A**) Individual scaled Gaussian distributions making up the modelled polysome profile. (**B**) The summed Gaussian distributions with added initial debris peak and baseline drift (the contribution of the latter two is shown as the broken grey line). (**C**) Overlay of the trace from panel B with the experimental polysome data (black dots) used to generate the fit.

Individual peaks in polysome profiles are reminiscent in shape of the bell-shaped curves of Gaussian (normal) distributions. We found that individual peaks of a profile fit Gaussian distributions well if the latter are adjusted for the width of individual peaks, and are multiplied with appropriate scaling factors to account for the peaks’ non-uniform size. Moreover, complete profiles can be closely approximated by summing appropriately scaled Gaussians at each of the pre-determined peak locations (Figure 3). In principle this follows the general approach of Gaussian mixture models (GMMs (38)), although our pre-determined peak locations constrain the model much more than is typically the case for GMMs. Importantly, the close fit between experimental and modelled polysomes extends throughout the whole gradient profile, including the higher polysome region where individual peaks are no longer distinguishable by eye. The good fit between experimental data and Gaussian models holds for polysome profiles derived from both actively translating yeast (Figure 3) and mammalian cell lines (Supplemental Figure S2).

### Modelling polysome profiles from ribosome footprinting data

Central parameters of modelled polysome profiles are the scaling factors for individual peaks. The area under an unscaled Gaussian distribution is one by definition, and in consequence these scaling factors are directly proportional to the contributions individual peaks make to the overall profile. In turn, the contribution of individual peaks to the profile is proportional to the amount of RNA present in the n-some population of transcripts represented by that peak. As discussed above, rRNA absorbance dominates the absorbance from other RNAs and we therefore approximate peak areas as being proportional to the number of ribosomes involved in the corresponding n-somes, neglecting contributions from mRNA and tRNA.

**Figure 4.**
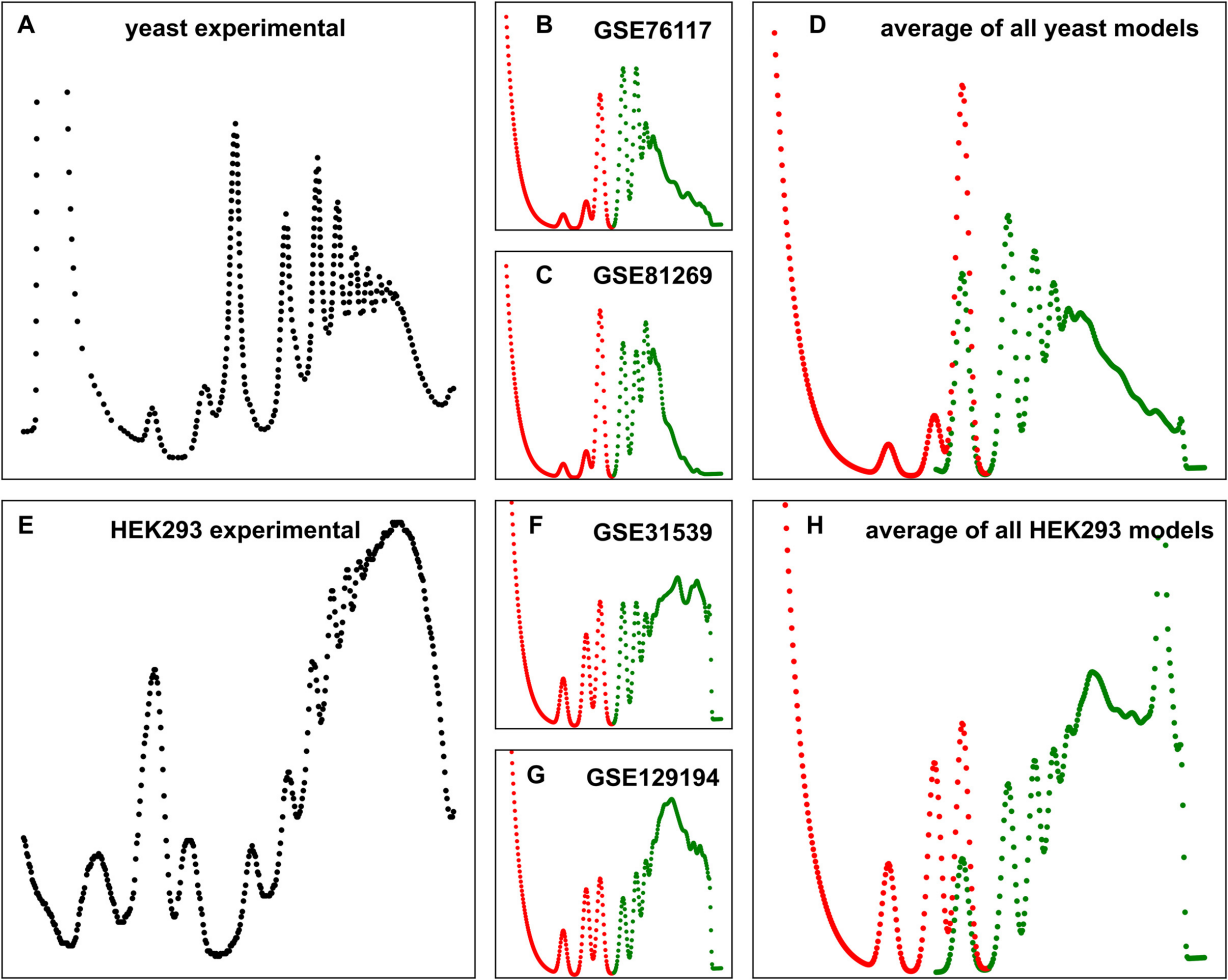
Comparison of experimental polysome profiles with profiles modelled from ribosome footprinting datasets. All datasets analysed in this figure represent control conditions where genetically wild-type cells were grown at optimal growth temperatures and in complete growth media. (**A**), representative experimental yeast profile. (**B, C**) Modelled polysome profiles generated from individual yeast footprinting datasets. (**D**) A modelled polysome profile generated from the average information in 37 yeast datasets used in this study. (**E**) Representative experimental dataset generated with cultured HEK293 cells. (**F,G**) Modelled polysome profiles generated from individual HEK293 datasets. (**H**) A modelled polysome profile generated from the average of seven HEK293 datasets analysed in this study. All coloured profiles show peaks generated exclusively from information in footprinting datasets in green, and peaks where additional information on the proportion of free ribosomal subunits contributed in red. In panels D and H, 80S peaks showing only translating monosomes (green) or the total monosome population modelled assuming 15% non-translating ribosomes (red) are shown overlaid for comparison.

The relationship between the number of ribosomes per transcript and scaling factors for individual peaks can be exploited to compute apparent polysome profiles from ribosome footprinting data. The general workflow for generating modelled polysome profiles is outlined in Supplemental Figure S3. Unlike real polysome profiles, modelled profiles lack information on non-translating ribosomes so that the monosome, 60S and 40S peaks cannot be fully determined in the latter. However, information on the proportion of inactive ribosomes in the cell as well as the proportion of inactive ribosomes split into their separate 40S and 60S subunits can be used to complete modelled profiles. In fast growing yeast, the proportion of inactive ribosomes has been experimentally estimated as 15% (31), and fitting to experimental profiles suggests that about 30% of these inactive ribosomes split into separate subunits. In contrast, mammalian HEK293 cells show a higher proportion of non-translating ribosomes split into separate subunits judging by the relatively higher peaks for the 40S and 60S subunits (Figure 4).

The application of the polysome modelling workflow to Ribo-Seq datasets reported for yeast and HEK293 cells is shown (Figure 4). For clarity, parts of the polysomes solely predicted by sequencing data are shown in green, whereas parts to which auxiliary data like the proportion of inactive ribosomes contribute are shown in red. For the 80S peak, two versions are shown in panels D and H of Figure 4, contrasting peaks modelled with (red) or without (green) inclusion of non-translating ribosomes.

Polysome profiles from different types of cells show distinct features owing to different translation dynamics. Yeast cells show a much less pronounced population of mRNAs with high ribosome content than HEK293 cells, with experimental yeast profiles typically showing a profile maximum around the tri-some peak (Figure 4A) whereas the mammalian cells show a profile maximum later in the gradient (Figure 4E). These distinctive features are preserved both in polysome models generated with selected individual datasets (Figure 4 B, C, F, G), as well as in the averaged models generated with larger numbers of datasets (Figure 4D, H). This demonstrates that polysome profiles modelled from Ribo-Seq datasets can faithfully present distinctive features observed in experimental polysome profiles for the same cell type.

### Visual assessment of published footprinting datasets using modelled polysome profiles

The modelling workflow was applied to 37 published yeast ribosome footprinting datasets (for details see Supplemental Table S1), all of which were generated as control experiments within their respective studies with untreated or parental yeast strains grown at 30°C and in YPD or Synthetic Complete medium. Thus, we expect these datasets to report approximately comparable translational activity profiles. Where RNA-Seq data were reported alongside the Ribo-Seq experiments, these were used to generate RNA copy number information, but where no such data were provided, typical transcript abundances were calculated from a reference RNA-Seq dataset (see below).

**Figure 5.**
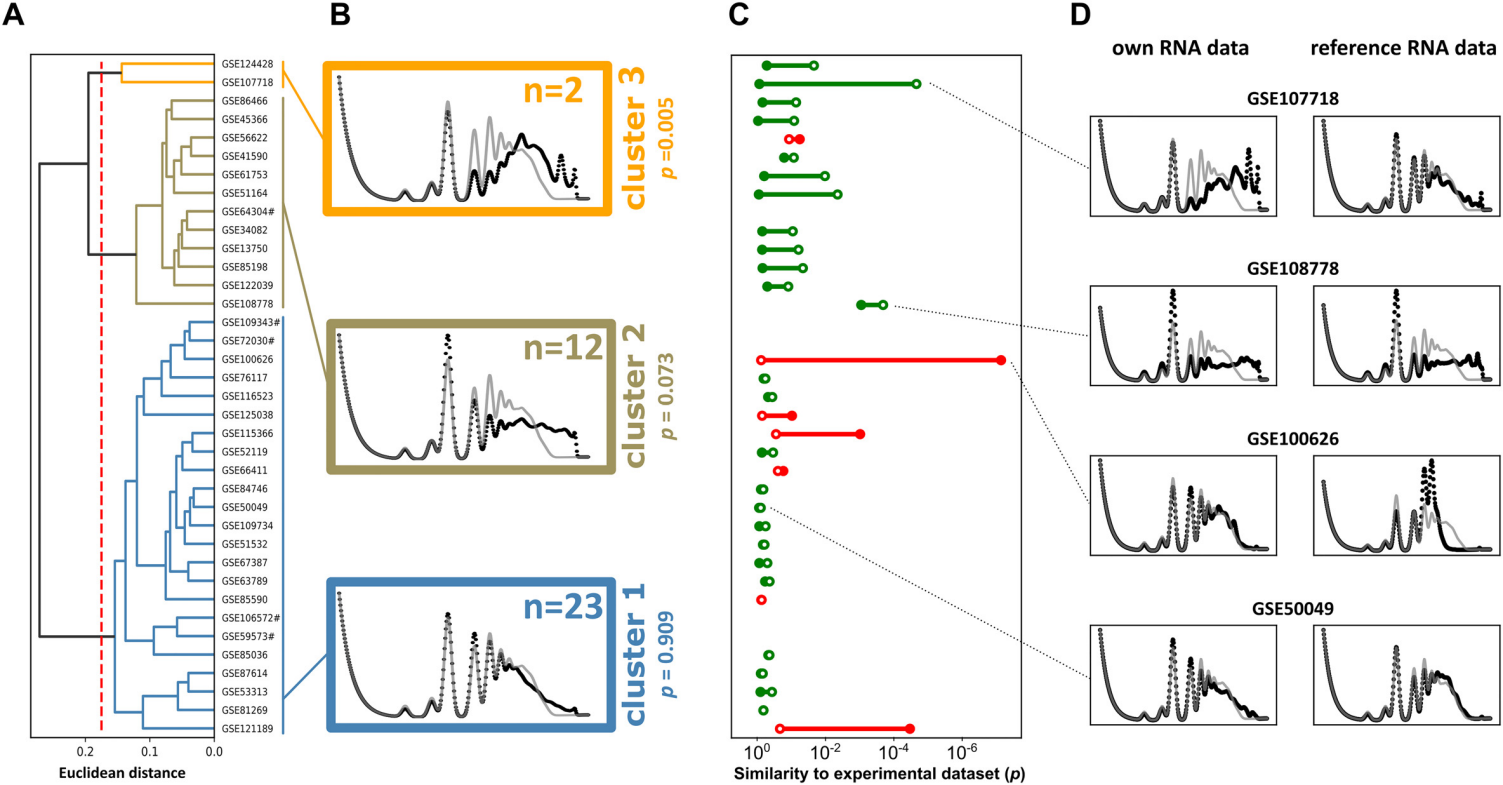
Comparison of modelled polysome profiles from 37 published yeast ribosome footprinting datasets. (**A**) Hierarchical clustering based on modelled polysome profiles was used to group datasets by similarity. The dashed red line indicates the similarity threshold used to define specific clusters. Polysome profiles were modelled based on the RNA data accompanying the Ribo-Seq datasets, except for datasets indicated by # where no RNA-Seq data were provided. (**B**) Meta polysomes (averaged polysome profiles) for each of the three clusters identified in (A) are represented as black dots, grey lines represent the experimental profile for comparison. The *P*-values evaluate the root mean square deviation (RMSD) in peak volumes between two datasets, and give the probability of observing a similar RMSD in comparisons between two *known good* datasets. (**C**) Visualization of the change in modelled polysomes when accompanying RNA-Seq data were replaced with a reference RNA-Seq dataset (see text for discussion). The graph shows the RMSD-derived *P*-values for comparison with the experimental dataset obtained when polysomes were modelled using the accompanying RNA-Seq data (open circles) or the reference data (closed circles). Colour indicates the direction of change, where green indicates that use of the reference data improves similarity to the experimental profile, and red indicates that similarity is worse with the reference data. (**D**) Plotted polysome profiles for selected pairs of analyses relying on own or reference RNA-Seq data. Black dots represent the modelled profiles, grey lines the experimental dataset for comparison.

While many of the investigated datasets resulted in modelled polysome profiles that closely resemble an experimental reference profile, this was not the case for all datasets (individual modelled profiles for all yeast datasets are presented in Supplemental Figure S4). To visualise differences between the generated profiles efficiently, we used a hierarchical clustering approach to define three clusters where the similarity to other profiles within the cluster was greater than similarity to profiles in other clusters (Figure 5A, B). Interestingly, this analysis revealed heterogeneity in the relative content of very highly ribosome-associated mRNAs in the datasets. In the three clusters, the modelled content of high polysomes increases with each cluster, with a corresponding decrease in low polysomes.

We sought to develop a quantitative measure that could support the qualitative visual interpretation of differences between modelled polysome profiles. In modelled profiles the peak volumes are normalised to the total area under the curve, making the root mean square deviation (RMSD) a useful measure for quantifying differences between profiles. When the 37 modelled yeast profiles were compared to the experimental reference profile, RMSD values ranged from 0.006 to 0.04. To make these numbers more intuitively interpretable, we derived an RMSD-based *P*-value, defined as the probability of observing a particular RMSD value in all pairwise comparisons for a reference *known good* collection of datasets (see the Materials and Methods section and analyses below for details). Figure 5B shows the corresponding *p-*values for the meta-profiles for each cluster in comparison to the experimental yeast profile, thereby quantitatively supporting the visual analysis that the difference between modelled polysome profiles and the experimental dataset increase with increasing cluster number.

Previous studies have shown that analyses of ribosome footprinting data can be skewed by biases in the accompanying RNA-Seq datasets (39). For example, poly(A)-selection can systematically bias against RNAs that are more prone to nuclease attack or physical breaking, or that have shorter poly(A) tails. Because poly(A) selection cannot be applied to Ribo-Seq samples, in combined RNA-Seq/Ribo-Seq experiments this could reduce the apparent abundance of such RNAs while correctly reporting the numbers of ribosomes bound to them, thereby reporting non-physiologically high ribosome loads. When we calculated apparent ribosome loads in datasets by dividing the number of ribosome protected fragments per transcript in the Ribo-Seq data by the abundance of that transcript in the accompanying RNA-Seq data, we observed that datasets in clusters 2 and 3, in contrast to those in cluster 1, reported very high and frequently physically impossible ribosome loads (Supplemental Figure S5). This indicates that distinct biases between RNA- and Ribo-Seq data may be one of the reasons leading to the apparent excess of heavy polysomes in some datasets.

To further investigate these findings, we established a reference dataset using a small number of RNA-Seq datasets from studies yielding modelled profiles closely matching experimental ones. In an iterative process, we selected datasets with an RMSD to the experimental profile below 0.02 (corresponding to RMSD-derived *P*-values above 0.1) that also showed the most consistent RNA expression levels between them (correlation coefficients > 0.7). When we re-modelled polysome profiles with this new RNA reference dataset, the similarity to the experimental data improved substantially in many cases (Figure 5C). Interestingly, for a small number of datasets (seven out of 37) the reference decreased the similarity between modelled and experimental profiles, although for three of these the change was negligible. Where the reference dataset increased the similarity between modelled and experimental profiles, it did so specifically by adjusting the heavy polysome content of the profiles (Figure 5D), indicating that the underlying cause for excess heavy polysomes in the modelled profiles is frequently associated with the RNA-Seq part of the datasets. When we re-clustered all modelled polysomes based on the new reference RNA-Seq dataset and applied the same clustering threshold as in Figure 5A, 32 of the 37 datasets formed a single cluster of profiles that were similar to the experimental dataset, whereas five datasets still showed profiles with clear differences to experimental data (Supplemental Figure S7). Of these five separately clustering datasets, two still yielded modelled profiles with an excess of heavy polysomes, whereas three yielded modelled profiles that appeared depleted for heavy polysomes.

We further validated that most ribosome profiles give biologically meaningful data when analysed with an improved reference RNA-Seq dataset by mapping where datasets reported transcripts with strong physical constraints on ribosome occupancy. This analysis revealed that both *RPL41* (a very short transcript where ribosome occupancy is limited by ORF length) and *GCN4* (a known translationally repressed transcript) are correctly reported as having very low ribosome densities, whereas longer and actively translated ORFs are reported as being more strongly ribosome associated (Supplemental Figure S6). Aside from validating that most Ribo-Seq datasets yield data consistent with known biological properties of transcripts when analysed with sound RNA-Seq datasets, this analysis also validates the data analysis pipeline we use for modelling the polysome profiles.

**Figure 6.**
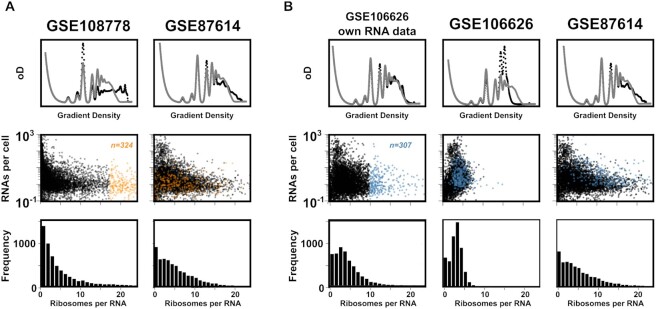
Correlation of transcript-specific ribosome densities between ribosome footprinting datasets. All data represent analyses with the reference RNA-Seq dataset except for the first column in panel B where RNA-Seq data accompanying the Ribo-Seq dataset were used. The top panels show modelled polysome profiles (black) with an experimental profile (grey) shown for comparison. Middle, RNA abundances plotted against ribosome densities for individual transcripts. Bottom, binned frequencies of ribosome densities for individual genes. (**A**), GSE108778 is a dataset displaying an apparent excess of heavy polysomes. The dataset contains a population of transcripts with low abundance but high ribosome loads (coloured orange). In GSE87614, a reference dataset where modelled polysomes show a high degree of similarity to an experimental profile, the same population of RNAs shows much lower ribosome densities. (**B**), GSE106626 is a dataset showing a well fitting modelled polysome profile when analysed with its accompanying RNA-Seq dataset, but a poorer fit when analysed with the reference RNA-Seq dataset. The 307 transcripts with the highest ribosome load (coloured in blue) correspond to a population that is also relatively ribosome dense in GSE87614, but that appear much less ribosome dense when GSE106626 is analysed with the reference dataset. The most parsimonious explanation for this behaviour is a balanced transcript bias in both the RNA-Seq and Ribo-Seq data of this dataset.

To explore why some datasets yield modelled polysome profiles that differed from experimental ones even when analysed with the reference dataset, we traced the translational states of individual transcripts in two selected outlier datasets and compared them to a comparator dataset from the main cluster (GSE87614, Figure 6). For the selected dataset with excess heavy polysomes, we observed a higher number of transcripts in the highest ribosome density region than for the comparator dataset (orange data points in Figure 6A). In the comparator dataset, these same transcripts show much lower ribosome densities. Since in these analyses the Ribo-Seq data from each dataset were paired with the reference RNA-Seq dataset, these differences can clearly be attributed to the Ribo-Seq data and indicate that GSE108778 reports biased numbers of ribosome-protected fragments for at least some transcripts.

For a second dataset, GSE100626, which yielded a well matching modelled profile when analysed with its accompanying RNA-Seq dataset but apparent depletion of heavy polysomes when analysed with refence data, we observed similar transcript-specific ribosome density patterns as for the comparator when analysed with its own RNA-Seq data (Figure 6B). However, with the reference RNA dataset the entire population of transcripts shifts to a region of low ribosome density. The dynamics of this change are exemplified by the subset of 307 transcripts coloured in blue, which contain all transcripts with ribosome loads of ten or higher in the analysis relying on the datasets own RNA-Seq data. In the analysis with the reference RNA dataset, these transcripts shift to higher abundance and consequently lower ribosome loads (since the same number of ribosome footprints is now divided by a larger number of transcripts). In the comparator dataset analysed with the reference RNA-Seq data, the same subset of transcripts shows a broader distribution across the upper two thirds of the range of ribosome densities.

Given that GSE100626 uses a strain background (SK1) and growth medium (YPD) that in other studies yields similar data to other strains, it is unlikely that these shifts reflect genuine biological gene expression differences. Instead, a more parsimonious explanation for these shifts is that in GSE100626 a subset of transcripts is represented lower than in the comparator in both the RNA-Seq and Ribo-Seq parts of the data. This balanced bias leads to a modelled polysome profile that appears physiological, but the bias is exposed when the profile is modelled with a less biased reference RNA-Seq dataset.

### Error sources in published footprinting datasets

**Figure 7.**
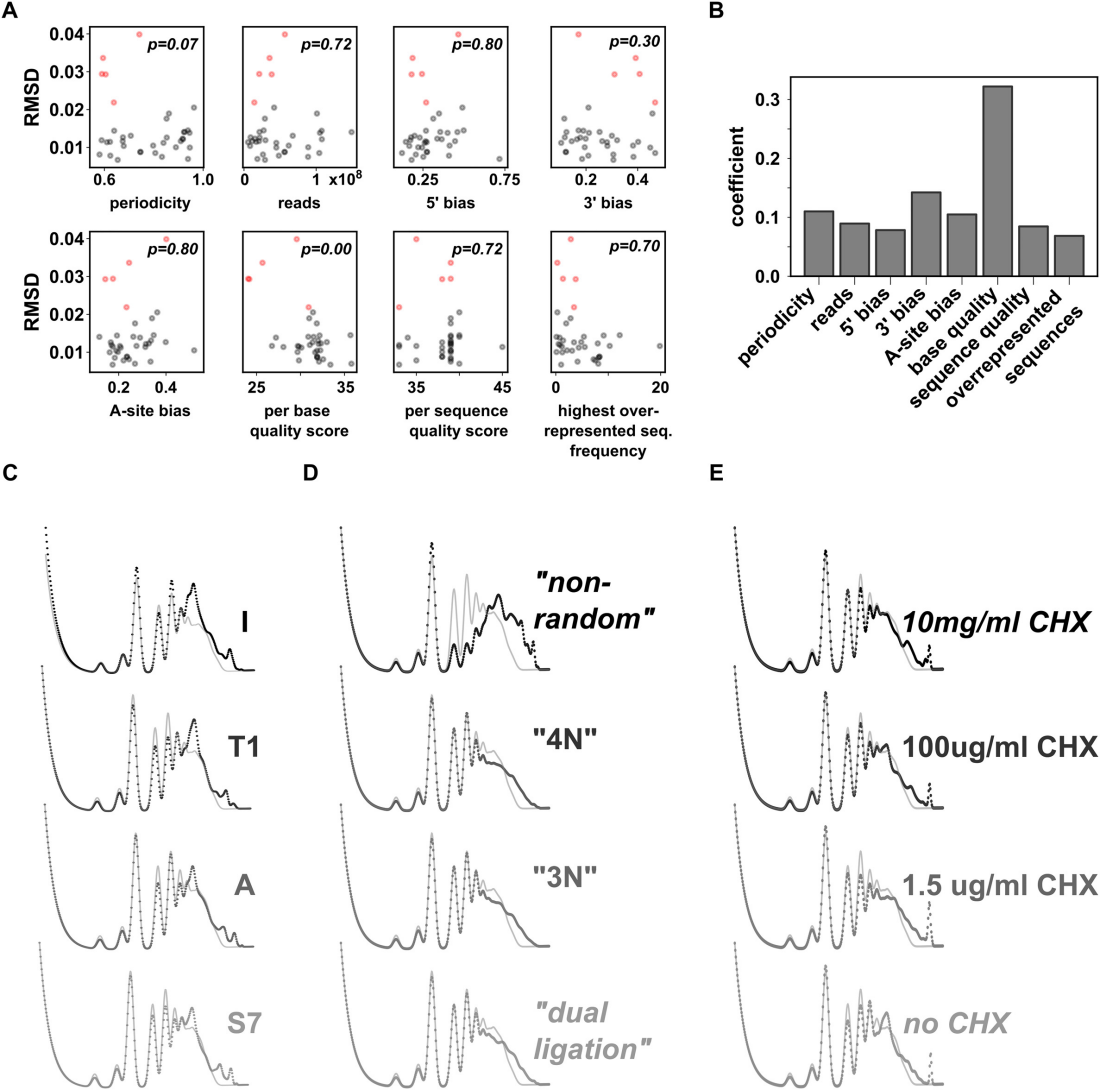
(A, B) The relationship between dataset quality and modelled polysome profiles in 37 yeast datasets. (**A**) Plots of dataset quality parameters against modelled profile similarity to an experimental profile (RMSD). Red datapoints identify the five datasets that cluster separately in Supplemental Figure S6, i.e. that still show differences to the experimental polysome profile when analysed with the reference RNA-Seq dataset. *p-*values show the significance of differences between red and grey datapoints (two-tailed *t*-test with Holm–Sidak multi-sample correction). (**B**) A decision tree was trained to predict datasets shown as red dots in panel A, using the complete set of dataset quality parameters as predictors. The contribution of each individual parameter to the trained model (‘coefficient’) is shown. (C–E) The apparent excess content of heavily ribosome associated transcripts is more sensitive to processes involved in fragment end formation than to cycloheximide concentrations. Modelled polysomes (back dots) are shown superimposed to an experimental polysome trace (grey lines) for datasets that generated polysome footprinting data using different nucleases (**C**) Different library preparation methods (**D**) or different cycloheximide concentrations (**E**) with otherwise invariant conditions. Changing the nature of the nuclease (C) or the library preparation method leads to stronger changes in apparent heavy polysome peaks than altering the cycloheximide concentration (E).

The results described above establish that modelled polysome profiles can be used as efficient visual aids to identify problematic ribosome footprinting datasets. The visual evaluation of modelled profiles can be aided by numerical measures such as the RMSD-derived *P*-values values for comparison between profiles. In additional analyses we aimed to determine which general dataset features establish the match between modelled and experimental polysome profiles. Datasets can be evaluated *via* different quality measures such as base and sequence quality-scores, read-depths and coverage. In addition, previous work (35) showed that the nature of ribosome protected mRNA fragments in different datasets displays different biases with respect to information in the A-site (which is expected), but also with respect to the sequence at the fragment 5′- and 3′-ends (which is likely related to differing nuclease efficiency in different sequence contexts). We assembled a context dataset that gathered both standard quality measures and measures of biases in ribosome-protected fragments, and analysed whether any of these quality features could predict the subset of datasets yielding poorly matching modelled polysome profiles when analysed with the reference mRNA data. We used two independent approaches for this, namely standard statistical tests (Figure 7A) and a decision-tree based approach (Extremely Randomised Trees (36), Figure 7B). Both approaches highlighted that the per-base quality score of the Ribo-Seq datasets is an important predictor, with low scores predicting a greater difference between modelled and experimental profiles. The next best predictors are periodicity and 3′-sequence bias of the ribosome protected fragments, although these two have much less predictive power than the per-base quality score. This analysis links features of modelled polysome profiles clearly to quality parameters of their originating Ribo-Seq datasets. It points to the importance of sequence quality for generating interpretable datasets, but also suggests that quality can be more subtly influenced by other experimental parameters.

Several studies have directly compared how different experimental parameters affect Ribo-Seq data, including nuclease selection (40), library preparation protocols (41) and cycloheximide concentrations (42). Nuclease selection and library preparation protocols directly affect some of the parameters related to sequence bias of ribosome protected fragments described in the previous paragraph, and we therefore asked whether different protocols would directly change the nature of the resulting modelled polysome profiles (Figure 7C–E).

With the published data using different nucleases to digest away non-ribosome protected RNA, modelled profiles changed subtly in their relative content of light and heavy polysome (Figure 7C). With datasets employing different library preparation methods, modelled polysome profiles were remarkably constant, with the exception of the ‘non-random’ method which led to a clear gain of heavy polysome content in the profile (Figure 7D). Both findings suggest that processes linked to fragment end formation can affect the quality of Ribo-Seq data, consistent with reported results (35) and with the limited association between RMSD values, 3′-fragment bias and periodicity observed above, and that at least some of these issues can be visualised by modelling polysome profiles from the datasets. Datasets using different cycloheximide concentrations also resulted in subtle changes (Figure 7E), although they showed no systematic pattern that correlated clearly with cycloheximide concentration. The observed changes may or may not therefore be actual results of the protocol modification.

### Polysome profiles from datasets generated under conditions of translational control

**Figure 8.**
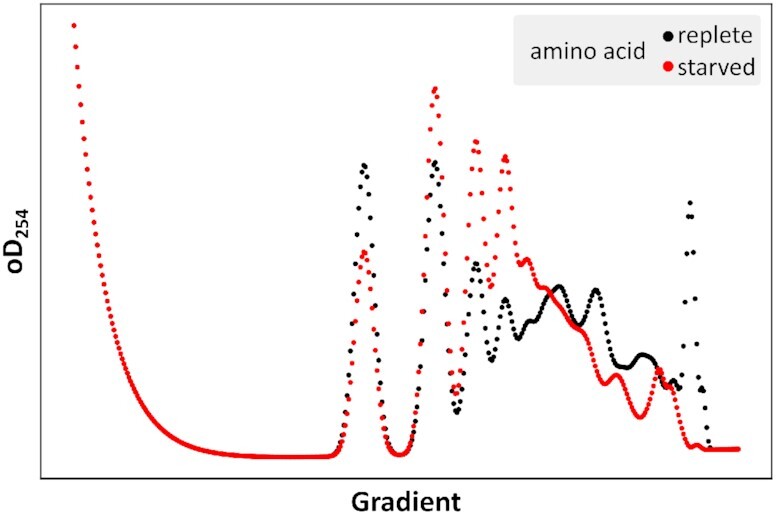
Comparison of modelled polysome profiles for Ribo-Seq datasets generated with cells grown in amino acid replete (black) or starved (red) conditions from GSE13750. Profiles were modelled without inclusion of non-translating ribosomes as discussed in the text.

We have so far discussed polysome modelling as a tool for assessing dataset integrity, by analysing Ribo-Seq datasets generated under well-defined control conditions where ancillary parameters such as the proportion of free ribosomes and transcript abundances are known, and where representative experimental datasets exist that can serve as reference for comparing to modelled profiles. It is also possible to use polysome modelling to visualise Ribo-Seq datasets generated under different non-control experimental conditions where translation is regulated, but this either requires determining ancillary parameters for such datasets, or restricting analyses to those aspects of the modelled polysomes for which ancillary parameters are not required. We illustrate this by comparing profiles for the Ribo-Seq datasets generated by Ingolia *et al.* (11) under amino acid replete and starvation conditions side-by-side (Figure 8). Both Ribo-Seq datasets are analysed with their accompanying RNA-Seq datasets, which for the replete condition leads to some excess heavy polysome content as discussed above (the dataset for this study is GSE13750). Because we do not know the proportion of free ribosomes during amino acid starvation, the polysomes in Figure 8 are modelled for both conditions without taking free ribosomes into account. Despite this limitation, significant transfer of ribosomes from heavier to lighter fractions is observed, consistent with the strong initiation block caused by amino acid starvation. One hallmark of strong initiation blocks is the transfer of ribosomes from the translating into the non-translating pool which is readily visible in experimental polysome profiles by a strongly enlarged 80S peak during amino acid starvation, illustrated for example in Figure 1 in Costello *et al.* (43). This transfer of ribosomes into the 80S pool is not apparent in the modelled profiles because non-translating ribosomes are not captured by the Ribo-Seq approach. Importantly, this is a consequence of the Ribo-Seq method which is simply reflected in (but not caused by) our polysome modelling approach. Although we have not explored this in detail, where experimental polysome profiles are available the comparison between modelled and experimental profiles would allow inferring some parameters of interest such as the proportion of ribosomes transferred into the non-translating state.

In summary, when data from ribosome footprinting datasets are projected into modelled polysome profiles, these agree well with profiles from independent sucrose density gradient analyses, suggesting that both approaches yield consistent information on the translational state of cells. While this validates both approaches in general, a subset of ribosome footprinting datasets suggest unphysiological levels of transcripts with high ribosome loads. In most cases this is appears to be the result of under-reported transcripts in the RNA-Seq data accompanying the Ribo-Seq datasets, whereas for a small number of cases this reflects issues in the Ribo-Seq data themselves. We link such issues to the quality of the reported sequences, as well as diverse experimental parameters including the end-formation processes that give rise to the ribosome-protected fragments. The visualisation of datasets as modelled polysome profiles provides a convenient means for the rapid visual assessment of dataset integrity and can reveal issues that would otherwise only emerge through in depth data analyses.

## DISCUSSION

The unprecedented detail with which ribosome footprinting reveals transcriptome-wide translational activity has revolutionised our understanding of translational control. Despite the great power of this approach, various technical aspects continue to be debated including the use of cycloheximide (15,44), library preparation methods (16,17) and statistical approaches to evaluating data (20,45,46). Moreover, for a subset of problems addressed in footprinting studies, the data appear to be variable between related studies. An example of this are codon decoding times, which have been addressed by various studies in different organisms without a clear and consistent picture emerging. This naturally raises questions over where such variability comes from.

The initial motivation for our study was to investigate to what degree information in footprinting data reflects information in polysome profiles, a longer established approach that gives simpler and generally well understood read-outs. By computationally reducing the information content of footprinting data to that of polysome profiles and then visualising this information in the familiar format of experimental polysome profile traces, we show that patterns of translation revealed by both approaches are consistent in principle (Figure 4). This further validates both techniques as approaches for assaying transcriptome-wide ribosome densities. However, our analyses also reveal clear heterogeneity between published ribosome footprinting datasets, especially regarding the nature of heavily ribosome-associated transcripts.

Since no absolute reference exists that could be used to assign a ‘correct’ polysome profile, it is not possible to unambiguously decide from this comparison alone which of the datasets is the most physiologically relevant. For example, heavy polysomes might be somehow obscured in experimental profiles due to the strong compression of peaks in the denser parts of the gradient, in which case modelled profiles with higher content of heavy polysomes might be more physiologically relevant. However, our additional analyses suggest that datasets displaying an apparent excess of heavy polysomes show additional and unambiguous artefacts, such as a high proportion of ribosome densities per transcript that exceed the theoretical upper limits of one ribosome every 10 codons (Supplemental Figure S5). In contrast, the datasets where modelled polysome profiles are most consistent with experimental profiles show the lowest proportion of transcripts with unphysiological ribosome loads. This suggests that experimental polysome profiles reflect the actual physiological transcriptome, and that footprinting datasets most consistent with experimental polysome profiles likely also reflect physiological translation.

Ribo-Seq has been extensively critiqued as a technique, and existing studies have pointed to potential artefacts in Ribo-Seq datasets such as changes in the relative flux of ribosomes along different parts of transcripts (18) or have highlighted how distorting influences of strong ribosomal pause sites affect the interpretation of Ribo-Seq data at nucleotide resolution (19). Consequently the interpretation of such data at very high resolution remains challenging (20). The approaches leading to these insights required the development or application of complex statistical methodology (20), and they primarily focus on methods for translating Ribo-Seq data into biological insights. Our method differs from these previous approaches in that it is based on a different set of assumptions about the data (namely that ribosome occupancies on transcripts are equally represented in Ribo-Seq and polysome profiling approaches), and in that we use the projection of Ribo-Seq data into a familiar visual format (rather than complex statistical approaches) as the principal means for evaluation. It also focuses on dataset integrity, rather than approaches for drawing conclusion from the datasets. We believe that the ability to assess datasets rapidly using modelled polysomes profiles as a visual tool will be a useful basis for the routine quality control of ribosome footprinting analyses, and would be very complementary when employed jointly with the robust analysis tools developed by others. We have demonstrated the validity of this approach for a simple eukaryotic model (yeast) and for mammalian cells, but we anticipate that the approach will work equally for all single-cell based eukaryotic systems that yield robust polysome profiles. It is unlikely to work for prokaryotic cells because the coupled transcription and translation reactions physically link transcripts with different ribosome densities.

The software providing the functionality for generating modelled profiles and for calculating the RMSD-derived *P-*values for profile comparisons is available as a convenient python package distributed via the Python Package Index (PyPI, https://pypi.org/project/polyan/), and we provide detailed instructions for its use via protocols.io (https://www.protocols.io/view/using-polyan-a-python-package-for-modelling-polyso-5jyl8mz28g2w). The decision as to when a ribosome footprinting dataset should be regarded with caution is to some degree subject to context and it is therefore difficult to provide clear advice in this respect. However, a reasonable strategy based on the results shown above would be to assess new datasets by visualising them both with their own RNA-Seq data (if available) as well as the reference dataset which is provided as part of the software; and calculating *P*-values against a ‘known good’ dataset which is equally provided as part of the software or which may be generated by the user. We would suggest that whenever *P*-values thus calculated approach 0.05, further and more detailed investigations as to how the dataset differs from the ‘known good’ reference is advisable.

In summary, our analyses show that many ribosome footprinting datasets agree well with translational activity patterns suggested by independent sucrose density gradient analyses. While this validates the ribosome footprinting approach in general, some ribosome footprinting datasets suggest non-physiologically high ribosome association of transcripts which contradicts evidence from both other datasets generated under comparable conditions, and from sucrose density gradients. The processes that lead to end formation in DNA fragment libraries may be one contributing source that can lead to such artefacts. Modelled polysome profiles provide a convenient means for the rapid visual assessment of data integrity in polysome footprinting datasets which can reveal some, although not all, such artefacts.

## DATA AVAILABILITY

All raw data and analysis scripts used in this study are available from the GitHub repository (https://github.com/tobiasvonderhaar/polysomes). The polysome modelling software is available as a Python package installable via the Python Package Index, PyPI (https://pypi.org/project/polyan/).

## Supplementary Material

gkac705_Supplemental_File
