# Supplementary material for: Evaluating data integrity in ribosome footprinting datasets through modelled polysome profiles
Source: Nucleic Acids Res. 2022 Aug 18;50(19):e112. doi: 10.1093/nar/gkac705 (PMC9638929; doi:10.1093/nar/gkac705)
Supplement: gkac705_Supplemental_File [file gkac705_supplemental_file.pdf]

Supplemental Materials for "Modelled polysome profiles reveal heterogeneity in published ribosome footprinting experiments"

Fabio Hedayioglu, Emma J. Mead, Owen J. Sansom, Giovanna R. Mallucci, Anne E Willis, C. Mark Smales and Tobias von der Haar

1 Peak distances in a HEK293 polysome profile

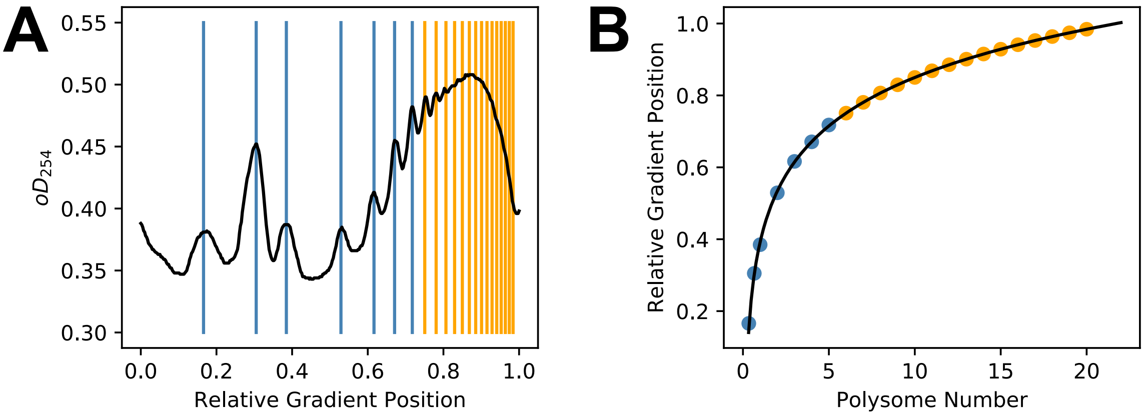

Supplemental Figure 1. Peak locations in polysomal gradients. This figure corresponds to figure 2 in the main manuscript except that the polysome profile in question was prepared from HEK293 cells grown to 70% confluence. (**a**) Peak locations in HEK293 polysome profiles. Blue lines indicate peak locations detected directly using a peak finding algorithm, orange lines indicate peak locations extrapolated from the directly detected peak locations. (**b**) Peak locations (coloured dots, colours corresponding to panel a) compared to the prediction function (black line).

2 Modelling a HEK293 polysome profile

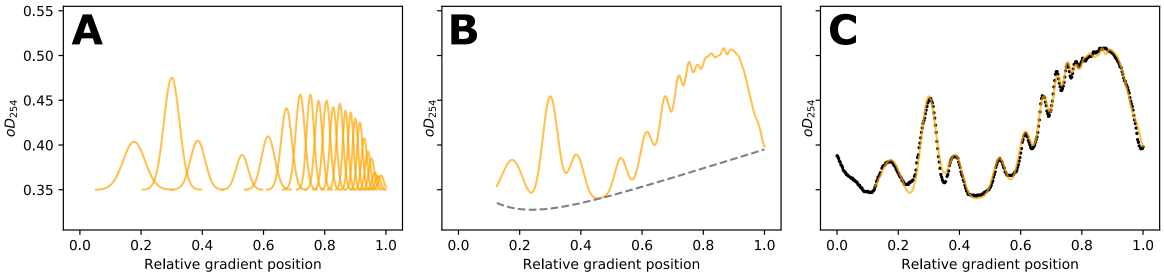

Supplemental figure 2. Approximation of a HEK293 polysomal gradient profile as a Gaussian mixture model. This figure corresponds to figure 3 in the main manuscript except that Gaussians are fitted to the HEK293 profile shown in supplemental figure 1 above. (**A**), individual scaled Gaussian distributions making up the modelled polysome profile, (**B**), the summed Gaussian distributions with added initial debris peak and baseline drift (the contribution of the latter two is shown as the broken grey line), (**C**), overlay of the trace from panel **B** with the experimental polysome data (black dots) used to generate the fit.

3 Modelling polysome profiles from experimental Ribo-Seq data

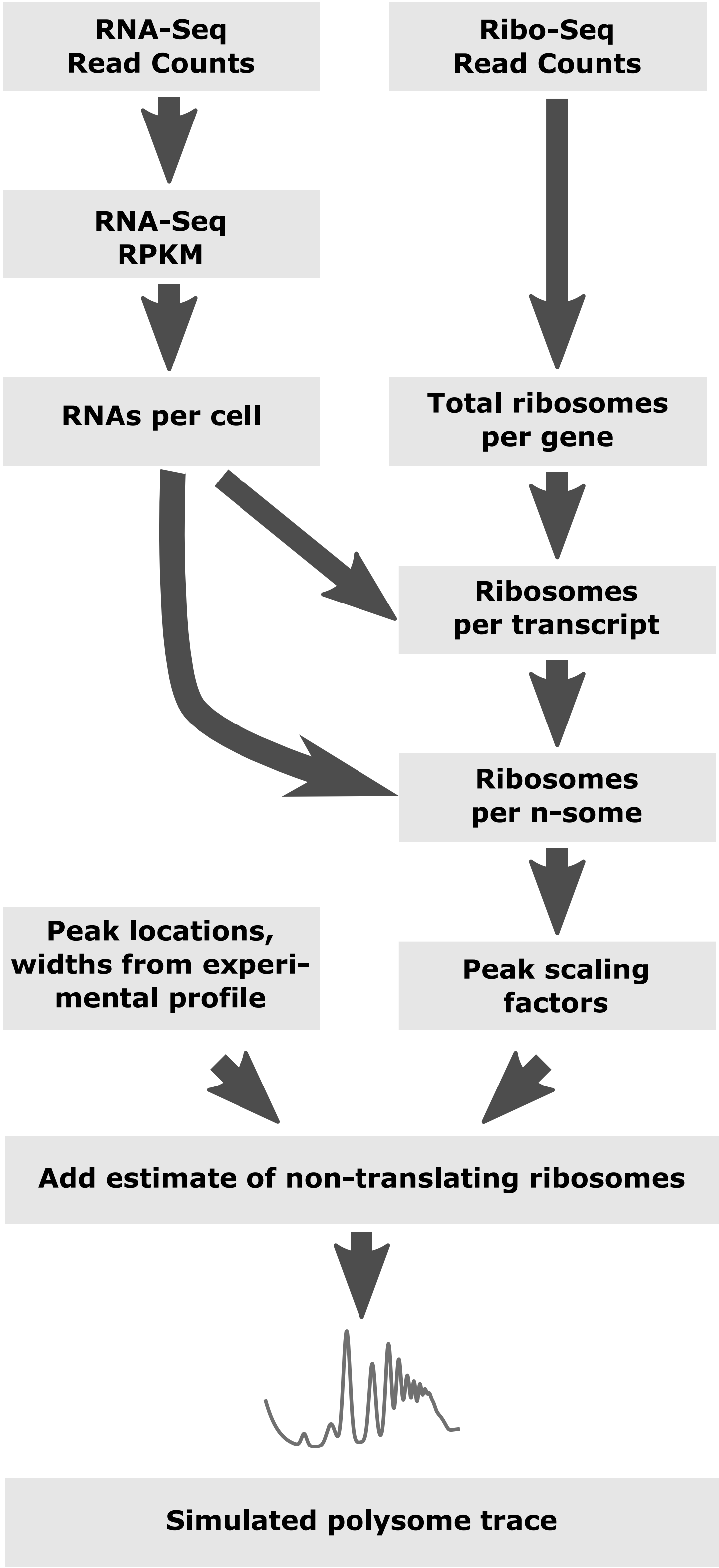

Supplemental Figure 3. Workflow for computing polysome profiles from ribosome footprinting data.

4 Summary of datasets

Series IDs and other details of datasets retrieved from GEO [1] are shown in supplemental table 1. Individual reconstructed polysome traces for these datasets are shown in supplemental figure 4. The "Cluster No" column identifies the cluster in figure 4 in the main text.

Supplemental table 1. Summary of yeast datasets used in this study.

GEO SeriesID	Strain	mRNA-Seq incl.	Temp	Medium	CHX addition[a]	Cluster No	Publication
GSE13750	BY4741	Yes	30	YPD	B	2	[1]
GSE34082	SK1	Yes	30	YPD	B	2	[2]
GSE41590	74D-694	Yes	30	YPD	B	2	[3]
GSE45366	BY4742	Yes	30	YPD	B	2	[4]
GSE50049	BY4716	Yes	30	YPD	B	1	[5]
GSE51164	BY4741	Yes	30	YPD	B	2	[6]
GSE51532	Σ1278b	Yes	30	YPD	B	1	[7]
GSE52119	S288C	Yes	30	YPD	B	1	[8]
GSE53313	ns	Yes	ns	ns	B	1	[9]
GSE56622	BY4741	Yes	30	SCD	B	2	[10]
GSE59573	BY4741	No	30	YPD	B	1	[11]
GSE61753	Σ1278b	Yes	30	YPD	B	2	[12]
GSE63789	S288C	Yes	30	SCD	N	1	[13]
GSE64304	Y7092	No	30	SC-Ura	B	2	[14]
GSE66411	BY4741	Yes	30	YPD	B	1	[15]
GSE67387	BY4741	Yes	30	YPD	B	1	[16]
GSE72030	BY4742	No	30	YPD	B	1	[17]
GSE76117	BY4741	Yes	30	YPD	B	1	[18]
GSE81269	BY4741	Yes	30	YPD	B	1	[19]
GSE84746	ns	Yes	ns	ns	A	1	[20]
GSE85036	BY4741	Yes	30	SC-Ura	A	1	[21]
GSE85198	BY4741	Yes	30	YPD	A	2	[22]
GSE85590	BY4741	Yes	30	YPD	B	1	[23]
GSE86466	W303-1A	Yes	30	YPD	B	2	[24]
GSE87614	BY4741	Yes	30	YPD	B	1	[25]
GSE100626	BY4741	Yes	30	YPD	B	1	[26]
GSE106572	BY4741	No	30	YPD	N	1	[27]
GSE107718	BY4741	Yes	30	YPD	B	3	[28]
GSE108778	SK1	Yes	30	YPD	B	2	[29]
GSE109343	Σ1278b	No	30	YPD	B	1	[30]
GSE109734	BY25598	Yes	30	YPD	B	1	[31]
GSE115366	SK1	Yes	30	YPD	B	1	[32]
GSE116523	S288C	Yes	30	YPD	A	1	[33]
GSE121189	SK1	Yes	30	YPD	A	1	[34]
GSE122039	BY4741	Yes	30	YPD	B	2	[35]
GSE124428	CEN.PK	Yes	30	SCD	A	3	[36]
GSE125038	BY4741	Yes	30	SCD	B	1	[37]

[a] B; Before harvest, A; After Harvest (during lysis); N, no CHX added. "ns", not stated in publication.

[1] http://www.ncbi.nlm.nih.gov/geo

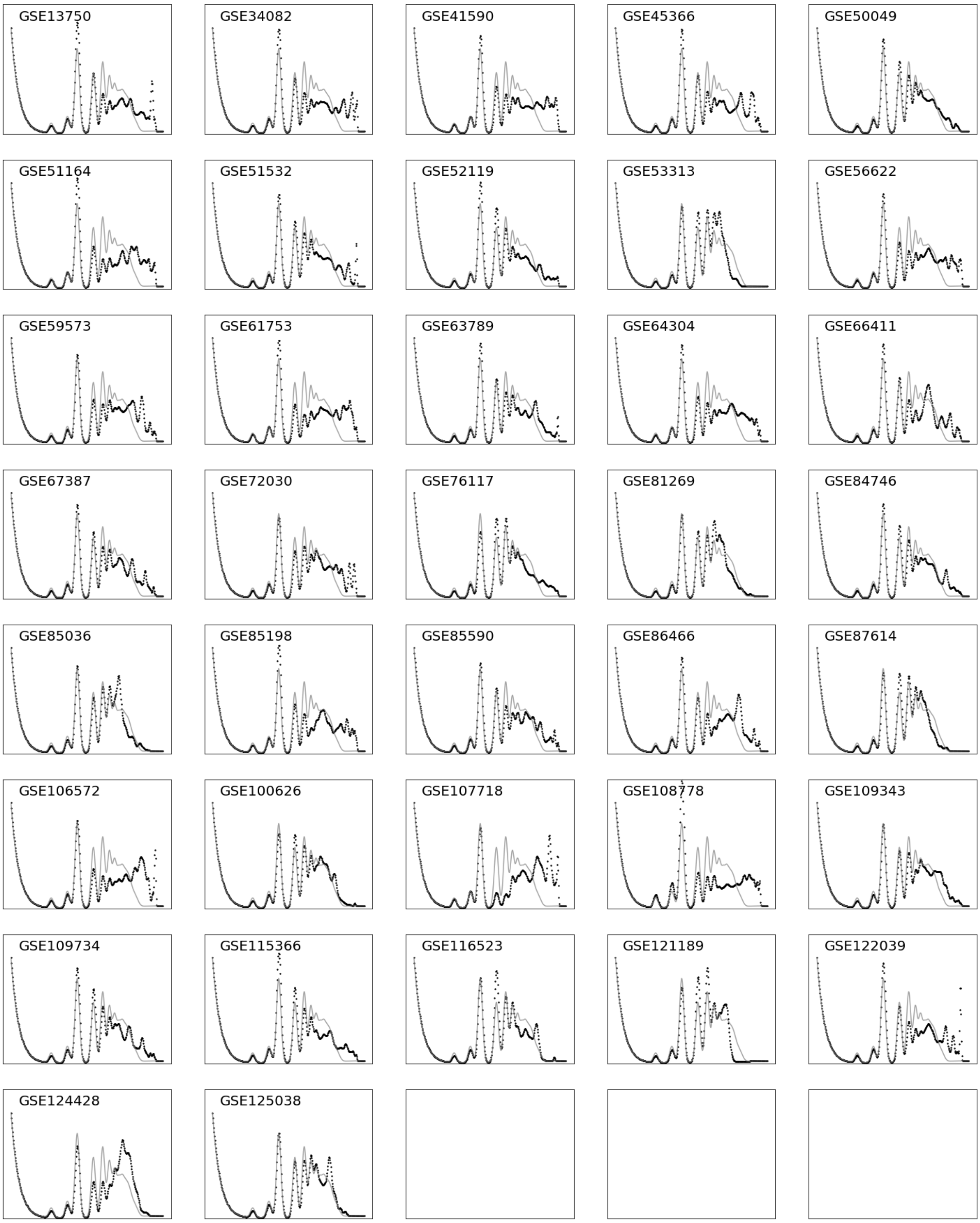

Supplemental figure 4. Modelled polysome profile of individual yeast datasets used in this study. Modelled profiles are shown as black dots, and are overlaid to the same experimental reference profile that is used in other figures in this manuscript.

5 Ribosome load per RNA

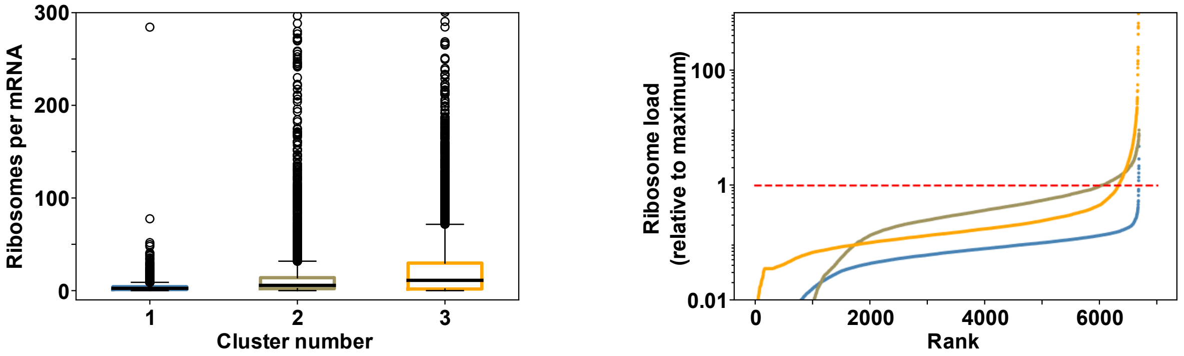

Supplemental figure 5. Comparing ribosome loads for the three dataset clusters identified by polysome modelling. The left panel shows ribosome *loading* per mRNA for the accumulated datasets in each cluster. The right panel shows ribosome *densities*, normalised to the theoretical maximum ribosome density of 1 ribosome every 10 codons. Data points above the red line represent transcripts that appear to show physically impossible ribosome densities.

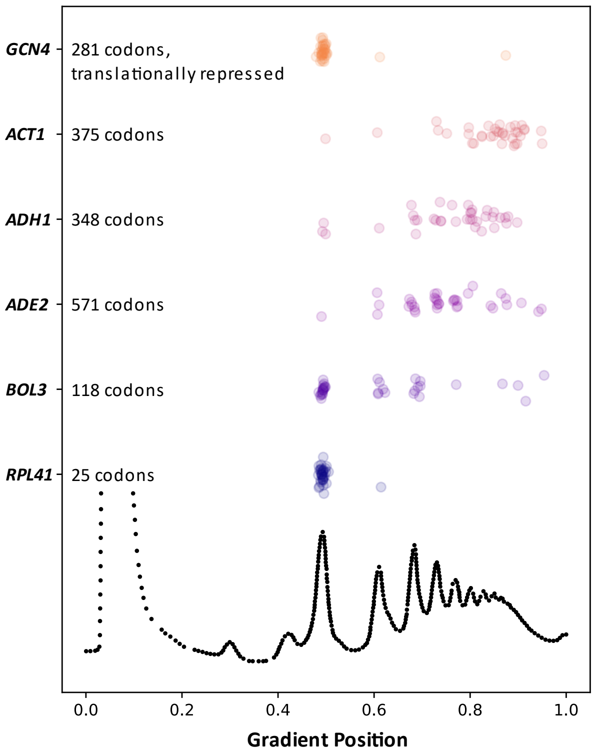

Supplemental figure 6. Peak locations for individual transcripts are consistent with known ORF length and translational control restrictions. Using the same data processing rules as during reconstruction of the polysome profiles, we investigated whether studies appeared to report physiologically possible ribosome occupancies for individual transcripts. Dots indicate the location of the peak containing the majority of transcripts in each of the 37 analysed studies. For very short transcripts like *RPL41* where ribosome occupancy is physically restricted to one or two ribosomes, all studies report peak densities within the physical occupancy limit. Similarly, transcripts known to be translationally restricted (*GCN4*) show low ribosome occupancy. Longer transcripts known to be actively translated show ribosome occupancy that increases as a function of translational activity and ORF length.

6 Clustering of polysomes modelled with a reference mRNA set

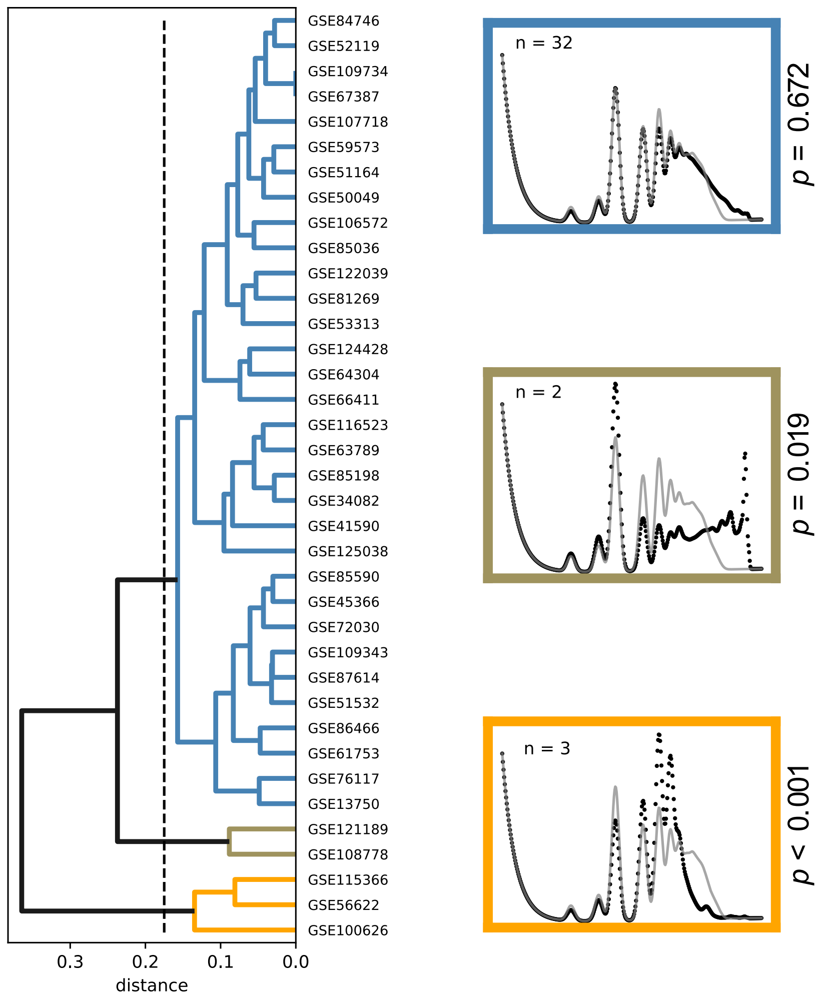

Supplemental figure 7. Datasets re-clustered as in figure 4 but using the reference RNA-Seq dataset. Left, dendrogram showing the results of hierarchical clustering of modelled polysomes, with identical parameters as for figure 4 in the main text but here for profiles modelled only with the reference RNA dataset. Right, averaged polysome models for the three cluster identified by colour in the dendrogram. *p*-values give the probability of observing the similarity between the averaged modelled and experimental profiles within a reference *known good* collection of datasets (see main text for explanation).

7 Supplemental material References

1. Ingolia NT, Ghaemmaghami S, Newman JRS, Weissman JS. Genome-Wide Analysis in Vivo of Translation with Nucleotide Resolution Using Ribosome Profiling. Science (80-). 2009;324: 218-223.

2. Brar GA, Yassour M, Friedman N, Regev A, Ingolia NT, Weissman JS. High-resolution view of the yeast meiotic program revealed by ribosome profiling. Science (80-). 2012;335: 552–557. doi:10.1126/science.1215110

3. Baudin-Baillieu A, Legendre R, Kuchly C, Hatin I, Demais S, Mestdagh C, et al. Genome-wide translational changes induced by the prion [PSI+]. Cell Rep. 2014;8: 439–48. doi:10.1016/j.celrep.2014.06.036

4. Zinshteyn B, Gilbert W V. Loss of a Conserved tRNA Anticodon Modification Perturbs Cellular Signaling. PLoS Genet. 2013;9: e1003675. doi:10.1371/journal.pgen.1003675

5. Artieri CG, Fraser HB. Evolution at two levels of gene expression in yeast. Genome Res. 2014;24: 411–421. doi:10.1101/gr.165522.113

6. Cai Y, Futcher B. Effects of the yeast RNA-binding protein Whi3 on the half-life and abundance of CLN3 mRNA and other targets. PLoS One. 2013;8: e84630. doi:10.1371/journal.pone.0084630

7. Vaidyanathan PP, Zinshteyn B, Thompson MK, Gilbert W V. Protein kinase A regulates gene-specific translational adaptation in differentiating yeast. RNA. 2014;20: 912–22. doi:10.1261/rna.044552.114

8. McManus CJ, May GE, Spealman P, Shteyman A. Ribosome profiling reveals post-transcriptional

buffering of divergent gene expression in yeast. Genome Res. 2014;24: 422–30. doi:10.1101/gr.164996.113

9. Subtelny AO, Eichhorn SW, Chen GR, Sive H, Bartel DP. Poly(A)-tail profiling reveals an embryonic switch in translational control. Nature. 2014;508: 66–71. doi:10.1038/nature13007

10. Zid BM, O'Shea EK. Promoter sequences direct cytoplasmic localization and translation of mRNAs during starvation in yeast. Nature. 2014;514: 117–121. doi:10.1038/nature13578

11. Kaya A, Gerashchenko M V, Seim I, Labarre J, Toledano MB, Gladyshev VN. Adaptive aneuploidy protects against thiol peroxidase deficiency by increasing respiration via key mitochondrial proteins. Proc Natl Acad Sci U S A. 2015;112: 10685–10690. doi:10.1073/pnas.1505315112

12. Thompson MK, Rojas-Duran MF, Gangaramani P, Gilbert W V. The ribosomal protein Asc1/RACK1 is required for efficient translation of short mRNAs. Elife. 2016;5. doi:10.7554/eLife.11154

13. Pop C, Rouskin S, Ingolia NT, Han L, Phizicky EM, Weissman JS, et al. Causal signals between codon bias, mRNA structure, and the efficiency of translation and elongation. Mol Syst Biol. 2014;10: 770–770. doi:10.15252/msb.20145524

14. Dhungel N, Eleuteri S, Li L-B, Kramer NJ, Chartron JW, Spencer B, et al. Parkinson's disease genes VPS35 and EIF4G1 interact genetically and converge on α-synuclein. Neuron. 2015;85: 76–87. doi:10.1016/j.neuron.2014.11.027

15. Sen ND, Zhou F, Ingolia NT, Hinnebusch AG. Genome-wide analysis of translational efficiency reveals distinct but overlapping functions of yeast DEAD-box RNA helicases Ded1 and eIF4A. Genome Res. 2015;25: 1196–205. doi:10.1101/gr.191601.115

16. Nedialkova DD, Leidel SA. Optimization of Codon Translation Rates via tRNA Modifications Maintains Proteome Integrity. Cell. The Authors; 2015;161: 1–13. doi:10.1016/j.cell.2015.05.022

17. Thiaville PC, Legendre R, Rojas-Benítez D, Baudin-Baillieu A, Hatin I, Chalancon G, et al. Global translational impacts of the loss of the tRNA modification t6A in yeast. Microb Cell. 2016;3: 29–45. doi:10.15698/mic2016.01.473

18. Heyer EE, Moore MJ. Redefining the Translational Status of 80S Monosomes. Cell. 2016;164: 757–769. doi:10.1016/j.cell.2016.01.003

19. Radhakrishnan A, Chen Y-H, Martin S, Alhusaini N, Green R, Coller J. The DEAD-Box Protein Dhh1p Couples mRNA Decay and Translation by Monitoring Codon Optimality. Cell. 2016;167: 122-132.e9. doi:10.1016/j.cell.2016.08.053

20. Lecanda A, Nilges BS, Sharma P, Nedialkova DD, Schwarz J, Vaquerizas JM, et al. Dual randomization of oligonucleotides to reduce the bias in ribosome-profiling libraries. Methods. 2016;107: 89–97. doi:10.1016/j.ymeth.2016.07.011

21. Ishikawa K, Makanae K, Iwasaki S, Ingolia NT, Moriya H. Post-Translational Dosage Compensation Buffers Genetic Perturbations to Stoichiometry of Protein Complexes. PLoS Genet. 2017;13: e1006554. doi:10.1371/journal.pgen.1006554

22. Beaupere C, Wasko BM, Lorusso J, Kennedy BK, Kaeberlein M, Labunskyy VM. CAN1 Arginine Permease Deficiency Extends Yeast Replicative Lifespan via Translational Activation of Stress Response Genes. Cell Rep. 2017;18: 1884–1892. doi:10.1016/j.celrep.2017.01.077

23. Mittal N, Guimaraes JC, Gross T, Schmidt A, Vina-Vilaseca A, Nedialkova DD, et al. The Gcn4 transcription factor reduces protein synthesis capacity and extends yeast lifespan. Nat Commun. 2017;8: 457. doi:10.1038/s41467-017-00539-y

24. Dörfel MJ, Fang H, Crain J, Klingener M, Weiser J, Lyon GJ. Proteomic and genomic characterization of a yeast model for Ogden syndrome. Yeast. 2017;34: 19–37. doi:10.1002/yea.3211

25. Zinshteyn B, Rojas-Duran MF, Gilbert W V. Translation initiation factor eIF4G1 preferentially binds yeast transcript leaders containing conserved oligo-uridine motifs. RNA. 2017;23: 1365–1375.

doi:10.1261/rna.062059.117

26. Chou H-J, Donnard E, Gustafsson HT, Garber M, Rando OJ. Transcriptome-wide Analysis of Roles for tRNA Modifications in Translational Regulation. Mol Cell. 2017;68: 978-992.e4. doi:10.1016/j.molcel.2017.11.002

27. Tunney R, McGlincy NJ, Graham ME, Naddaf N, Pachter L, Lareau LF. Accurate design of translational output by a neural network model of ribosome distribution. Nat Struct Mol Biol. 2018;25: 577–582. doi:10.1038/s41594-018-0080-2

28. Diament A, Feldman A, Schochet E, Kupiec M, Arava Y, Tuller T. The extent of ribosome queuing in budding yeast. PLoS Comput Biol. 2018;14: e1005951. doi:10.1371/journal.pcbi.1005951

29. Cheng Z, Otto GM, Powers EN, Keskin A, Mertins P, Carr SA, et al. Pervasive, Coordinated Protein-Level Changes Driven by Transcript Isoform Switching during Meiosis. Cell. 2018;172: 910-923.e16. doi:10.1016/j.cell.2018.01.035

30. Wang YJ, Vaidyanathan PP, Rojas-Duran MF, Udeshi ND, Bartoli KM, Carr SA, et al. Lso2 is a conserved ribosome-bound protein required for translational recovery in yeast. PLoS Biol. 2018;16: e2005903. doi:10.1371/journal.pbio.2005903

31. Blasco-Moreno B, de Campos-Mata L, Böttcher R, García-Martínez J, Jungfleisch J, Nedialkova DD, et al. The exonuclease Xrn1 activates transcription and translation of mRNAs encoding membrane proteins. Nat Commun. 2019;10: 1298. doi:10.1038/s41467-019-09199-6

32. Van Dalfsen KM, Hodapp S, Keskin A, Otto GM, Berdan CA, Higdon A, et al. Global Proteome Remodeling during ER Stress Involves Hac1-Driven Expression of Long Undecoded Transcript Isoforms. Dev Cell. 2018;46: 219-235.e8. doi:10.1016/j.devcel.2018.06.016

33. Sharma AK, Sormanni P, Ahmed N, Ciryam P, Friedrich UA, Kramer G, et al. A chemical kinetic basis for measuring translation initiation and elongation rates from ribosome profiling data. PLoS Comput Biol. 2019;15: e1007070. doi:10.1371/journal.pcbi.1007070

34. Cheng Z, Mugler CF, Keskin A, Hodapp S, Chan LY-L, Weis K, et al. Small and Large Ribosomal Subunit Deficiencies Lead to Distinct Gene Expression Signatures that Reflect Cellular Growth Rate. Mol Cell. 2019;73: 36-47.e10. doi:10.1016/j.molcel.2018.10.032

35. Makeeva DS, Lando AS, Anisimova A, Egorov AA, Logacheva MD, Penin AA, et al. Translatome and transcriptome analysis of TMA20 (MCT-1) and TMA64 (eIF2D) knockout yeast strains. Data Br. 2019;23: 103701. doi:10.1016/j.dib.2019.103701

36. Gupta R, Walvekar AS, Liang S, Rashida Z, Shah P, Laxman S. A tRNA modification balances carbon and nitrogen metabolism by regulating phosphate homeostasis. Elife. 2019;8: e44795. doi:10.7554/eLife.44795

37. Santos DA, Shi L, Tu BP, Weissman JS. Cycloheximide can distort measurements of mRNA levels and translation efficiency. Nucleic Acids Res. 2019;47: 4974–4985. doi:10.1093/nar/gkz205
